# 1-[5-(2-Chloro­phen­yl)-5-hy­droxy-3-methyl-4,5-dihydro-1*H*-pyrazol-1-yl]­ethanone

**DOI:** 10.1107/S1600536812007283

**Published:** 2012-02-24

**Authors:** Sheng-Hai Guo, Ji-Liang Wang, Dong-Qiang Guo, Xin-Ying Zhang

**Affiliations:** a School of Chemistry and Environmental Science, Henan Key Laboratory for Environmental Pollution Control, Henan Normal University, Xinxiang, Henan 453007, People’s Republic of China

## Abstract

The title compound, C_12_H_13_ClN_2_O_2_, crystallizes with two independent but very similar mol­ecules (*A* and *B*) in the asymmetric unit. The pyrazole ring in each mol­ecule has an envelope conformation. The dihedral angle between the pyrazole ring mean plane and the benzene ring is 86.07 (14)° in *A* and 85.99 (14)° in *B*. In the crystal, the *A* and *B* mol­ecules are linked *via* a pair of O—H⋯O hydrogen bonds, forming dimers. These dimers are further linked *via* C—H⋯O inter­actions to form –*A*–*B*–*A*–*B*– chains propagating along the *c*-axis direction.

## Related literature

For the bioactivities of 5-hy­droxy­pyrazolines, see: Sauzem *et al.* (2008 ▶); Zhao *et al.* (2009 ▶); Idrees *et al.* (2009 ▶). For the crystal structures of related 5-hy­droxy­pyrazolines, see: Kargar, Kia, Froozandeh *et al.* (2011 ▶); Kargar, Kia, Moghadamm *et al.* (2011 ▶).
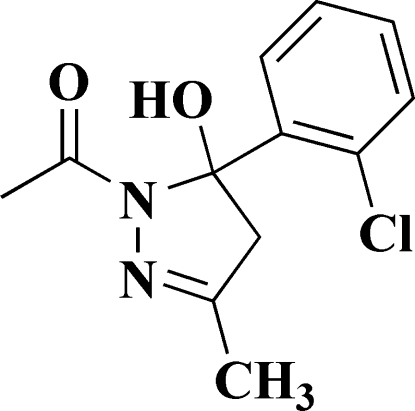


## Experimental

### 

#### Crystal data

C_12_H_13_ClN_2_O_2_
*M* *_r_* = 252.70
Monoclinic, 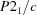

*a* = 10.320 (3) Å
*b* = 14.916 (4) Å
*c* = 16.346 (4) Å
β = 95.158 (3)°
*V* = 2506.0 (12) Å^3^
*Z* = 8
Mo *K*α radiation
μ = 0.30 mm^−1^
*T* = 296 K
0.39 × 0.25 × 0.15 mm

#### Data collection

Bruker SMART CCD area-detector diffractometer
Absorption correction: multi-scan (*SADABS*; Bruker, 2007 ▶) *T*_min_ = 0.893, *T*_max_ = 0.957
16979 measured reflections
4663 independent reflections
3077 reflections with *I* > 2σ(*I*)
*R*_int_ = 0.038

#### Refinement

*R*[*F* ^2^ > 2σ(*F* ^2^)] = 0.053
*wR*(*F* ^2^) = 0.159
*S* = 1.02
4663 reflections
313 parameters
H-atom parameters constrained
Δρ_max_ = 0.39 e Å^−3^
Δρ_min_ = −0.35 e Å^−3^

### 

Data collection: *SMART* (Bruker, 2007 ▶); cell refinement: *SAINT* (Bruker, 2007 ▶); data reduction: *SAINT*; program(s) used to solve structure: *SHELXS97* (Sheldrick, 2008 ▶); program(s) used to refine structure: *SHELXL97* (Sheldrick, 2008 ▶); molecular graphics: *SHELXTL* (Sheldrick, 2008 ▶); software used to prepare material for publication: *SHELXTL*.

## Supplementary Material

Crystal structure: contains datablock(s) I, global. DOI: 10.1107/S1600536812007283/su2378sup1.cif

Structure factors: contains datablock(s) I. DOI: 10.1107/S1600536812007283/su2378Isup2.hkl

Supplementary material file. DOI: 10.1107/S1600536812007283/su2378Isup3.cdx

Supplementary material file. DOI: 10.1107/S1600536812007283/su2378Isup4.cml

Additional supplementary materials:  crystallographic information; 3D view; checkCIF report

## Figures and Tables

**Table 1:** Hydrogen-bond geometry (Å, °)

*D*—H⋯*A*	*D*—H	H⋯*A*	*D*⋯*A*	*D*—H⋯*A*
O1—–H1⋯..O4^i^	0.82	1.97	2.748 (3)	159
O3—–H3*A*⋯..O2^ii^	0.82	2.03	2.792 (3)	155
C8—–H8*B*⋯..O3^iii^	0.97	2.53	3.410 (3)	151
C20—–H20*B*⋯..O1^iv^	0.97	2.50	3.354 (3)	147

Symmetry codes: (i) 
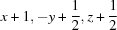
; (ii) 
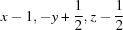
; (iii) 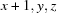; (iv) 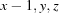.

**Fig. 1.**
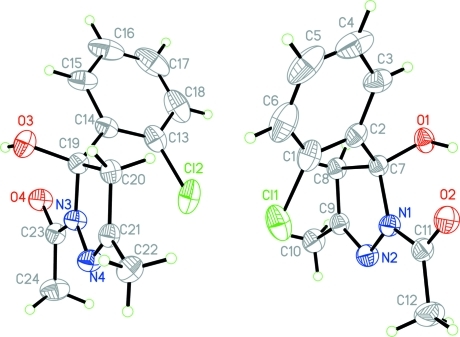
Molecular structure of the two independent molecules (A right; B left) of the title compound, with displacement ellipsoids drawn at the 30% probability level.

**Fig. 2.**
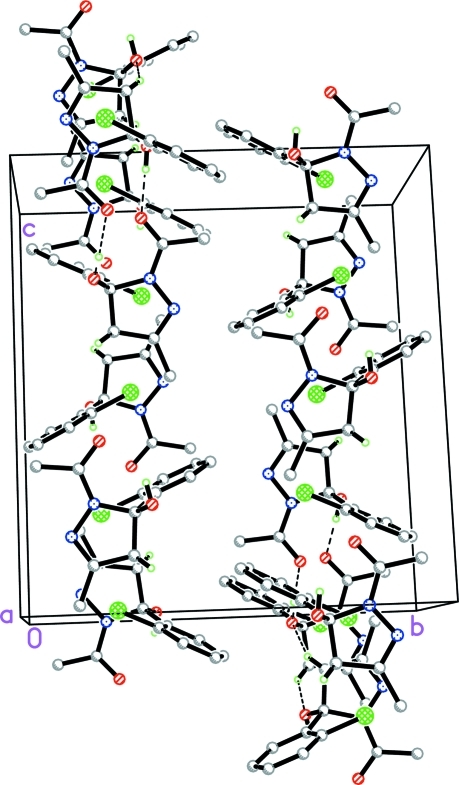
Crystal packing of the title compound, viewed along the a axis. The O—H···O hydrogen bonds and C-H···O interactions are shown as dashed lines (see Table 1 for details; H atoms not involved in these interactions have been omitted for clarity).

## supplementary crystallographic information

### Comment

5-Hydroxypyrazolines have drawn much attention due to their interesting biological properties such as anti-inflammatory, antibiotic, and hypolipidemic activities (Sauzem *et al.*, 2008; Zhao *et al.*, 2009; Idrees *et al.*, 2009). Crystal structures of some 5-hydroxypyrazoline derivatives have been reported (Kargar, Kia, Froozandeh *et al.*, 2011; Kargar, Kia, Moghadamm *et al.*, 2011). Herein, we report on the crystal structure of the new title 5-hydroxypyrazoline derivative.

The title compound crystallizes with two independent but very similar molecules (A and B) in the asymmetric unit (Fig. 1). All the bond lengths and bond angles are within normal ranges. The five-membered pyrazole rings have envelope conformations with atom C7 as the flap in molecule A, and atom C19 as the flap in molecule B. The dihedral angle between the pyrazole ring mean plane and the phenyl ring is 86.07 (14) ° in A and 85.99 (14) ° in B.

In the crystal, the A and B molecules are linked *via* a pair of O—H···O hydrogen bonds forming dimers. These dimers are further linked via C-H···O interactions to form -A-B-A-B- chains propagating along the c axis direction (Table 1 and Fig. 2).

### Experimental

1-(2-chlorophenyl)butane-1,3-dione (1.0 mmol), acetohydrazide (1.0 mmol), and a drop of concentrated H_2_SO_4_ were mixed and ground for 10 min in a mortar. Upon completion of the reaction, monitored by TLC, ethyl acetate and water were added to the reaction mixture. Then, the organic layer was washed with Na_2_CO_3_ solution and water, and dried over anhydrous Na_2_SO_4_. Ethyl acetate was removed under reduced pressure and the residue was purified by chromatography on silica-gel to provide the title product as a white solid. Colourless block-like crystals of the title compound, suitable for *X*-ray diffraction analysis, were obtained by slow evaporation of the solvent from a dichloromethane solution at room temperature.

### Refinement

The H atoms were included in calculated positions and were refined as riding atoms: O—H = 0.82 Å, and C—H = 0.93, 0.97, 0.96 Å for aromatic, methylene and methyl H atoms, respectively, with *U*_iso_(H) = k ×*U*_eq_(O,*C*), where k = 1.5 for OH and methyl H atoms, and *k* = 1.2 for all other H atoms.

### Crystal data

**Table:**

C_12_H_13_ClN_2_O_2_	*F*(000) = 1056
*M**_r_* = 252.70	*D*_x_ = 1.340 Mg m^−^^3^
Monoclinic, *P*2_1_/*c*	Mo *K*α radiation, λ = 0.71073 Å
Hall symbol: -P 2ybc	Cell parameters from 3669 reflections
*a* = 10.320 (3) Å	θ = 2.4–25.9°
*b* = 14.916 (4) Å	µ = 0.30 mm^−^^1^
*c* = 16.346 (4) Å	*T* = 296 K
β = 95.158 (3)°	Block, colourless
*V* = 2506.0 (12) Å^3^	0.39 × 0.25 × 0.15 mm
*Z* = 8	

### Data collection

**Table:**

Bruker SMART CCD area-detector diffractometer	4663 independent reflections
Radiation source: fine-focus sealed tube	3077 reflections with *I* > 2σ(*I*)
Graphite monochromator	*R*_int_ = 0.038
phi and ω scans	θ_max_ = 25.5°, θ_min_ = 2.4°
Absorption correction: multi-scan (*SADABS*; Bruker, 2007)	*h* = −12→12
*T*_min_ = 0.893, *T*_max_ = 0.957	*k* = −18→18
16979 measured reflections	*l* = −19→19

### Refinement

**Table:**

Refinement on *F*^2^	Primary atom site location: structure-invariant direct methods
Least-squares matrix: full	Secondary atom site location: difference Fourier map
*R*[*F*^2^ > 2σ(*F*^2^)] = 0.053	Hydrogen site location: inferred from neighbouring sites
*wR*(*F*^2^) = 0.159	H-atom parameters constrained
*S* = 1.02	*w* = 1/[σ^2^(*F*_o_^2^) + (0.0804*P*)^2^ + 0.8196*P*] where *P* = (*F*_o_^2^ + 2*F*_c_^2^)/3
4663 reflections	(Δ/σ)_max_ = 0.001
313 parameters	Δρ_max_ = 0.39 e Å^−^^3^
0 restraints	Δρ_min_ = −0.35 e Å^−^^3^

### Special details

**Table:**

Geometry. All e.s.d.'s (except the e.s.d. in the dihedral angle between two l.s. planes)are estimated using the full covariance matrix. The cell e.s.d.'s are takeninto account individually in the estimation of e.s.d.'s in distances, anglesand torsion angles; correlations between e.s.d.'s in cell parameters are onlyused when they are defined by crystal symmetry. An approximate (isotropic)treatment of cell e.s.d.'s is used for estimating e.s.d.'s involving l.s. planes.
Refinement. Refinement of *F*^2^ against ALL reflections. The weighted *R*-factor *wR* and goodness of fit *S* are based on *F*^2^, conventional *R*-factors *R* are based on *F*, with *F* set to zero for negative *F*^2^. The threshold expression of *F*^2^ > σ(*F*^2^) is used only for calculating *R*-factors(gt) *etc*. and is not relevant to the choice of reflections for refinement. *R*-factors based on *F*^2^ are statistically about twice as large as those based on *F*, and *R*- factors based on ALL data will be even larger.

### Fractional atomic coordinates and isotropic or equivalent isotropic displacement parameters (Å^2^)

**Table:**

	*x*	*y*	*z*	*U*_iso_*/*U*_eq_	
C1	0.5236 (3)	0.2945 (2)	0.27457 (16)	0.0630 (8)	
C2	0.6517 (3)	0.32278 (17)	0.26911 (14)	0.0484 (6)	
C3	0.6858 (3)	0.40628 (19)	0.30087 (17)	0.0643 (8)	
H3	0.7701	0.4272	0.2977	0.077*	
C4	0.5966 (5)	0.4597 (2)	0.3375 (2)	0.0904 (12)	
H4	0.6214	0.5154	0.3591	0.109*	
C5	0.4714 (5)	0.4294 (3)	0.3415 (2)	0.1025 (15)	
H5	0.4112	0.4653	0.3650	0.123*	
C6	0.4349 (4)	0.3468 (3)	0.3110 (2)	0.0891 (12)	
H6	0.3506	0.3261	0.3148	0.107*	
C7	0.7497 (2)	0.27082 (16)	0.22292 (14)	0.0427 (6)	
C8	0.7104 (2)	0.26559 (16)	0.12996 (14)	0.0452 (6)	
H8A	0.6204	0.2833	0.1172	0.054*	
H8B	0.7656	0.3035	0.0997	0.054*	
C9	0.7293 (2)	0.16951 (17)	0.11060 (15)	0.0470 (6)	
C10	0.7195 (3)	0.1336 (2)	0.02506 (16)	0.0630 (8)	
H10A	0.7276	0.0695	0.0267	0.094*	
H10B	0.7879	0.1585	−0.0040	0.094*	
H10C	0.6368	0.1497	−0.0026	0.094*	
C11	0.7751 (3)	0.14051 (18)	0.32065 (16)	0.0514 (6)	
C12	0.7807 (4)	0.0402 (2)	0.32863 (19)	0.0811 (10)	
H12A	0.8560	0.0181	0.3047	0.122*	
H12B	0.7038	0.0144	0.3006	0.122*	
H12C	0.7857	0.0240	0.3857	0.122*	
C13	0.3110 (3)	0.3263 (2)	−0.02266 (16)	0.0621 (8)	
C14	0.1813 (3)	0.34881 (17)	−0.01755 (14)	0.0470 (6)	
C15	0.1388 (3)	0.43010 (17)	−0.05224 (17)	0.0658 (8)	
H15	0.0529	0.4476	−0.0491	0.079*	
C16	0.2215 (5)	0.4854 (2)	−0.0913 (2)	0.0941 (13)	
H16	0.1904	0.5387	−0.1151	0.113*	
C17	0.3479 (5)	0.4620 (3)	−0.0950 (2)	0.1070 (15)	
H17	0.4032	0.4998	−0.1207	0.128*	
C18	0.3958 (4)	0.3819 (3)	−0.0606 (2)	0.0906 (12)	
H18	0.4825	0.3659	−0.0628	0.109*	
C19	0.0867 (2)	0.29137 (16)	0.02655 (14)	0.0427 (6)	
C20	0.1250 (2)	0.27892 (16)	0.11897 (14)	0.0459 (6)	
H20A	0.2142	0.2975	0.1335	0.055*	
H20B	0.0680	0.3128	0.1515	0.055*	
C21	0.1094 (2)	0.18061 (16)	0.13134 (15)	0.0457 (6)	
C22	0.1166 (3)	0.13570 (19)	0.21316 (15)	0.0564 (7)	
H22A	0.1115	0.0719	0.2057	0.085*	
H22B	0.0455	0.1555	0.2427	0.085*	
H22C	0.1974	0.1508	0.2438	0.085*	
C23	0.0645 (2)	0.16960 (16)	−0.07964 (15)	0.0455 (6)	
C24	0.0633 (3)	0.07144 (18)	−0.09644 (19)	0.0707 (9)	
H24A	0.0579	0.0615	−0.1547	0.106*	
H24B	−0.0106	0.0447	−0.0742	0.106*	
H24C	0.1417	0.0449	−0.0712	0.106*	
Cl1	0.46874 (8)	0.19108 (7)	0.23531 (5)	0.0841 (3)	
Cl2	0.37469 (8)	0.22580 (7)	0.01857 (5)	0.0865 (3)	
N1	0.7575 (2)	0.17426 (13)	0.24382 (12)	0.0457 (5)	
N2	0.7540 (2)	0.11902 (14)	0.17364 (13)	0.0522 (5)	
N3	0.0850 (2)	0.19699 (13)	−0.00094 (11)	0.0443 (5)	
N4	0.0897 (2)	0.13575 (14)	0.06455 (12)	0.0508 (5)	
O1	0.87374 (17)	0.30941 (12)	0.23266 (10)	0.0525 (5)	
H1	0.9088	0.2968	0.2782	0.079*	
O2	0.7846 (2)	0.19093 (13)	0.37965 (11)	0.0621 (5)	
O3	−0.03948 (17)	0.32648 (13)	0.01884 (10)	0.0548 (5)	
H3A	−0.0705	0.3216	−0.0289	0.082*	
O4	0.04921 (19)	0.22575 (12)	−0.13467 (10)	0.0562 (5)	

### Atomic displacement parameters (Å^2^)

**Table:**

	*U*^11^	*U*^22^	*U*^33^	*U*^12^	*U*^13^	*U*^23^
C1	0.0586 (18)	0.090 (2)	0.0407 (16)	0.0137 (15)	0.0055 (13)	0.0096 (14)
C2	0.0569 (16)	0.0557 (16)	0.0319 (13)	0.0083 (12)	0.0006 (11)	0.0080 (11)
C3	0.092 (2)	0.0540 (17)	0.0465 (16)	0.0113 (15)	0.0059 (15)	0.0048 (13)
C4	0.148 (4)	0.068 (2)	0.056 (2)	0.040 (2)	0.015 (2)	0.0058 (17)
C5	0.125 (4)	0.124 (4)	0.061 (2)	0.076 (3)	0.025 (2)	0.014 (2)
C6	0.072 (2)	0.133 (3)	0.064 (2)	0.037 (2)	0.0174 (17)	0.017 (2)
C7	0.0456 (14)	0.0472 (14)	0.0348 (13)	−0.0022 (10)	0.0012 (10)	0.0062 (10)
C8	0.0484 (14)	0.0532 (15)	0.0333 (13)	−0.0031 (11)	0.0005 (10)	0.0055 (11)
C9	0.0489 (15)	0.0547 (15)	0.0366 (13)	−0.0019 (11)	0.0000 (11)	0.0008 (11)
C10	0.077 (2)	0.0695 (18)	0.0415 (15)	0.0044 (15)	−0.0013 (14)	−0.0070 (13)
C11	0.0562 (16)	0.0556 (16)	0.0412 (15)	−0.0062 (12)	−0.0019 (12)	0.0097 (12)
C12	0.123 (3)	0.0602 (19)	0.0563 (18)	−0.0050 (18)	−0.0099 (19)	0.0154 (15)
C13	0.0633 (19)	0.084 (2)	0.0388 (15)	−0.0168 (15)	0.0021 (13)	−0.0014 (14)
C14	0.0620 (17)	0.0494 (15)	0.0295 (12)	−0.0092 (12)	0.0039 (11)	−0.0054 (10)
C15	0.103 (2)	0.0453 (16)	0.0503 (17)	−0.0065 (15)	0.0106 (16)	−0.0037 (13)
C16	0.161 (4)	0.057 (2)	0.067 (2)	−0.032 (2)	0.019 (2)	0.0005 (16)
C17	0.145 (4)	0.108 (3)	0.072 (2)	−0.068 (3)	0.029 (3)	0.000 (2)
C18	0.080 (2)	0.133 (3)	0.061 (2)	−0.039 (2)	0.0171 (18)	−0.007 (2)
C19	0.0475 (14)	0.0461 (13)	0.0340 (13)	0.0015 (10)	0.0010 (10)	−0.0024 (10)
C20	0.0494 (15)	0.0553 (15)	0.0323 (13)	−0.0012 (11)	0.0003 (11)	−0.0026 (11)
C21	0.0447 (14)	0.0539 (15)	0.0383 (14)	0.0022 (11)	0.0022 (11)	0.0031 (11)
C22	0.0665 (18)	0.0631 (17)	0.0398 (14)	0.0044 (14)	0.0052 (12)	0.0092 (12)
C23	0.0493 (15)	0.0513 (14)	0.0355 (13)	0.0007 (11)	0.0009 (11)	−0.0032 (11)
C24	0.109 (3)	0.0518 (17)	0.0513 (17)	0.0034 (16)	0.0051 (16)	−0.0105 (13)
Cl1	0.0578 (5)	0.1235 (8)	0.0712 (6)	−0.0265 (4)	0.0062 (4)	−0.0022 (5)
Cl2	0.0548 (5)	0.1328 (8)	0.0718 (6)	0.0203 (5)	0.0049 (4)	0.0159 (5)
N1	0.0562 (13)	0.0474 (12)	0.0329 (11)	−0.0004 (9)	−0.0002 (9)	0.0033 (9)
N2	0.0632 (14)	0.0517 (13)	0.0409 (12)	−0.0004 (10)	0.0007 (10)	−0.0042 (10)
N3	0.0593 (13)	0.0433 (11)	0.0297 (10)	−0.0032 (9)	0.0010 (9)	0.0015 (8)
N4	0.0657 (14)	0.0487 (12)	0.0375 (12)	−0.0013 (10)	0.0016 (10)	0.0055 (10)
O1	0.0500 (11)	0.0672 (12)	0.0394 (10)	−0.0108 (8)	−0.0015 (8)	0.0060 (8)
O2	0.0814 (14)	0.0671 (12)	0.0357 (10)	−0.0027 (10)	−0.0057 (9)	0.0036 (9)
O3	0.0541 (11)	0.0728 (12)	0.0372 (10)	0.0132 (9)	0.0018 (8)	−0.0012 (9)
O4	0.0762 (13)	0.0562 (11)	0.0345 (10)	−0.0032 (9)	−0.0052 (9)	0.0011 (8)

### Geometric parameters (Å, º)

**Table:**

C1—C6	1.378 (4)	C13—Cl2	1.748 (3)
C1—C2	1.398 (4)	C14—C15	1.393 (4)
C1—Cl1	1.746 (3)	C14—C19	1.527 (3)
C2—C3	1.383 (4)	C15—C16	1.383 (5)
C2—C7	1.527 (3)	C15—H15	0.9300
C3—C4	1.393 (5)	C16—C17	1.357 (6)
C3—H3	0.9300	C16—H16	0.9300
C4—C5	1.375 (6)	C17—C18	1.391 (6)
C4—H4	0.9300	C17—H17	0.9300
C5—C6	1.369 (6)	C18—H18	0.9300
C5—H5	0.9300	C19—O3	1.399 (3)
C6—H6	0.9300	C19—N3	1.477 (3)
C7—O1	1.400 (3)	C19—C20	1.538 (3)
C7—N1	1.481 (3)	C20—C21	1.491 (3)
C7—C8	1.539 (3)	C20—H20A	0.9700
C8—C9	1.484 (4)	C20—H20B	0.9700
C8—H8A	0.9700	C21—N4	1.281 (3)
C8—H8B	0.9700	C21—C22	1.492 (3)
C9—N2	1.283 (3)	C22—H22A	0.9600
C9—C10	1.492 (3)	C22—H22B	0.9600
C10—H10A	0.9600	C22—H22C	0.9600
C10—H10B	0.9600	C23—O4	1.229 (3)
C10—H10C	0.9600	C23—N3	1.348 (3)
C11—O2	1.220 (3)	C23—C24	1.490 (4)
C11—N1	1.350 (3)	C24—H24A	0.9600
C11—C12	1.503 (4)	C24—H24B	0.9600
C12—H12A	0.9600	C24—H24C	0.9600
C12—H12B	0.9600	N1—N2	1.410 (3)
C12—H12C	0.9600	N3—N4	1.405 (3)
C13—C14	1.389 (4)	O1—H1	0.8200
C13—C18	1.391 (4)	O3—H3A	0.8200
			
C6—C1—C2	121.7 (3)	C15—C14—C19	119.3 (3)
C6—C1—Cl1	116.9 (3)	C16—C15—C14	121.5 (4)
C2—C1—Cl1	121.3 (2)	C16—C15—H15	119.3
C3—C2—C1	117.2 (3)	C14—C15—H15	119.3
C3—C2—C7	119.0 (2)	C17—C16—C15	120.2 (4)
C1—C2—C7	123.4 (2)	C17—C16—H16	119.9
C2—C3—C4	121.4 (3)	C15—C16—H16	119.9
C2—C3—H3	119.3	C16—C17—C18	120.7 (3)
C4—C3—H3	119.3	C16—C17—H17	119.6
C5—C4—C3	119.5 (4)	C18—C17—H17	119.6
C5—C4—H4	120.3	C13—C18—C17	118.5 (4)
C3—C4—H4	120.3	C13—C18—H18	120.8
C6—C5—C4	120.5 (3)	C17—C18—H18	120.8
C6—C5—H5	119.7	O3—C19—N3	110.10 (19)
C4—C5—H5	119.7	O3—C19—C14	112.1 (2)
C5—C6—C1	119.6 (4)	N3—C19—C14	112.44 (19)
C5—C6—H6	120.2	O3—C19—C20	106.78 (19)
C1—C6—H6	120.2	N3—C19—C20	100.30 (18)
O1—C7—N1	110.09 (19)	C14—C19—C20	114.4 (2)
O1—C7—C2	112.0 (2)	C21—C20—C19	103.33 (19)
N1—C7—C2	113.86 (19)	C21—C20—H20A	111.1
O1—C7—C8	106.87 (19)	C19—C20—H20A	111.1
N1—C7—C8	100.51 (18)	C21—C20—H20B	111.1
C2—C7—C8	112.74 (19)	C19—C20—H20B	111.1
C9—C8—C7	103.38 (19)	H20A—C20—H20B	109.1
C9—C8—H8A	111.1	N4—C21—C20	114.1 (2)
C7—C8—H8A	111.1	N4—C21—C22	121.4 (2)
C9—C8—H8B	111.1	C20—C21—C22	124.5 (2)
C7—C8—H8B	111.1	C21—C22—H22A	109.5
H8A—C8—H8B	109.1	C21—C22—H22B	109.5
N2—C9—C8	114.6 (2)	H22A—C22—H22B	109.5
N2—C9—C10	122.3 (2)	C21—C22—H22C	109.5
C8—C9—C10	123.2 (2)	H22A—C22—H22C	109.5
C9—C10—H10A	109.5	H22B—C22—H22C	109.5
C9—C10—H10B	109.5	O4—C23—N3	119.4 (2)
H10A—C10—H10B	109.5	O4—C23—C24	122.5 (2)
C9—C10—H10C	109.5	N3—C23—C24	118.2 (2)
H10A—C10—H10C	109.5	C23—C24—H24A	109.5
H10B—C10—H10C	109.5	C23—C24—H24B	109.5
O2—C11—N1	120.0 (2)	H24A—C24—H24B	109.5
O2—C11—C12	123.1 (2)	C23—C24—H24C	109.5
N1—C11—C12	116.9 (2)	H24A—C24—H24C	109.5
C11—C12—H12A	109.5	H24B—C24—H24C	109.5
C11—C12—H12B	109.5	C11—N1—N2	122.0 (2)
H12A—C12—H12B	109.5	C11—N1—C7	125.3 (2)
C11—C12—H12C	109.5	N2—N1—C7	112.58 (18)
H12A—C12—H12C	109.5	C9—N2—N1	107.4 (2)
H12B—C12—H12C	109.5	C23—N3—N4	121.4 (2)
C14—C13—C18	122.1 (3)	C23—N3—C19	125.1 (2)
C14—C13—Cl2	121.0 (2)	N4—N3—C19	112.90 (18)
C18—C13—Cl2	116.9 (3)	C21—N4—N3	107.6 (2)
C13—C14—C15	117.1 (3)	C7—O1—H1	109.5
C13—C14—C19	123.6 (2)	C19—O3—H3A	109.5
			
C6—C1—C2—C3	−0.8 (4)	C15—C14—C19—N3	130.6 (2)
Cl1—C1—C2—C3	179.10 (19)	C13—C14—C19—C20	61.5 (3)
C6—C1—C2—C7	−174.6 (2)	C15—C14—C19—C20	−115.9 (3)
Cl1—C1—C2—C7	5.4 (3)	O3—C19—C20—C21	102.6 (2)
C1—C2—C3—C4	0.6 (4)	N3—C19—C20—C21	−12.3 (2)
C7—C2—C3—C4	174.6 (2)	C14—C19—C20—C21	−132.8 (2)
C2—C3—C4—C5	−0.7 (5)	C19—C20—C21—N4	9.7 (3)
C3—C4—C5—C6	1.1 (5)	C19—C20—C21—C22	−170.9 (2)
C4—C5—C6—C1	−1.4 (5)	O2—C11—N1—N2	176.0 (2)
C2—C1—C6—C5	1.2 (5)	C12—C11—N1—N2	−4.6 (4)
Cl1—C1—C6—C5	−178.7 (3)	O2—C11—N1—C7	0.7 (4)
C3—C2—C7—O1	11.4 (3)	C12—C11—N1—C7	−179.8 (3)
C1—C2—C7—O1	−175.0 (2)	O1—C7—N1—C11	75.2 (3)
C3—C2—C7—N1	137.2 (2)	C2—C7—N1—C11	−51.5 (3)
C1—C2—C7—N1	−49.2 (3)	C8—C7—N1—C11	−172.3 (2)
C3—C2—C7—C8	−109.1 (2)	O1—C7—N1—N2	−100.4 (2)
C1—C2—C7—C8	64.5 (3)	C2—C7—N1—N2	132.8 (2)
O1—C7—C8—C9	103.5 (2)	C8—C7—N1—N2	12.0 (2)
N1—C7—C8—C9	−11.4 (2)	C8—C9—N2—N1	−1.1 (3)
C2—C7—C8—C9	−133.0 (2)	C10—C9—N2—N1	179.5 (2)
C7—C8—C9—N2	8.5 (3)	C11—N1—N2—C9	176.7 (2)
C7—C8—C9—C10	−172.0 (2)	C7—N1—N2—C9	−7.5 (3)
C18—C13—C14—C15	0.0 (4)	O4—C23—N3—N4	173.4 (2)
Cl2—C13—C14—C15	−179.9 (2)	C24—C23—N3—N4	−7.5 (4)
C18—C13—C14—C19	−177.4 (3)	O4—C23—N3—C19	2.1 (4)
Cl2—C13—C14—C19	2.7 (3)	C24—C23—N3—C19	−178.8 (2)
C13—C14—C15—C16	1.0 (4)	O3—C19—N3—C23	72.1 (3)
C19—C14—C15—C16	178.6 (3)	C14—C19—N3—C23	−53.7 (3)
C14—C15—C16—C17	−1.4 (5)	C20—C19—N3—C23	−175.6 (2)
C15—C16—C17—C18	0.8 (6)	O3—C19—N3—N4	−99.7 (2)
C14—C13—C18—C17	−0.7 (5)	C14—C19—N3—N4	134.4 (2)
Cl2—C13—C18—C17	179.2 (3)	C20—C19—N3—N4	12.5 (3)
C16—C17—C18—C13	0.3 (6)	C20—C21—N4—N3	−1.9 (3)
C13—C14—C19—O3	−176.7 (2)	C22—C21—N4—N3	178.6 (2)
C15—C14—C19—O3	5.9 (3)	C23—N3—N4—C21	−179.6 (2)
C13—C14—C19—N3	−52.0 (3)	C19—N3—N4—C21	−7.4 (3)

### Hydrogen-bond geometry (Å, º)

**Table:**

*D*—H···*A*	*D*—H	H···*A*	*D*···*A*	*D*—H···*A*
O1—–H1···..O4^i^	0.82	1.97	2.748 (3)	159
O3—–H3*A*···..O2^ii^	0.82	2.03	2.792 (3)	155
C8—–H8*B*···..O3^iii^	0.97	2.53	3.410 (3)	151
C20—–H20*B*···..O1^iv^	0.97	2.50	3.354 (3)	147

Symmetry codes: (i) *x*+1, −*y*+1/2, *z*+1/2; (ii) *x*−1, −*y*+1/2, *z*−1/2; (iii) *x*+1, *y*, *z*; (iv) *x*−1, *y*, *z*.
